# Soil microbial communities shift in response to cropping sequence diversification with perennial seed crops

**DOI:** 10.3389/frmbi.2026.1808609

**Published:** 2026-05-26

**Authors:** Newton Z. Lupwayi, Nityananda Khanal, Mathew Richards, Rodrigo Ortega Polo

**Affiliations:** 1 Lethbridge Research and Development Centre, Agriculture and Agri-Food Canada, Lethbridge, AB, Canada; 2 Beaverlodge Research Farm, Agriculture and Agri-Food Canada, Beaverlodge, AB, Canada

**Keywords:** annual crops, crop rotation, nitrogen fertilizer, perennial crops, soil enzyme activities, soil health, soil microbial diversity

## Abstract

Integrating perennial forage seed crops into annual cropping sequences can diversify the rotations and improve soil health, yet their effects on the soil microbial communities and functions are not yet fully elucidated on the Canadian prairies. Using a 10-year field experiment with eight cropping sequences under varying supplemental nitrogen (N) fertilization levels, we evaluated the impacts of integrating perennial seed crops and annual crops on soil microbial biomass carbon (MBC), the composition and diversity of prokaryotic and fungal communities, and the activities of key enzymes involved in carbon (C), N, phosphorus (P), and sulfur (S) cycling, namely β-glucosidase, N-acetyl-β-glucosaminidase, acid phosphomonoesterase and arylsulfatase. The crop sequences containing intermittent succession of perennial and annual crops had 17% greater soil MBC, higher fungal richness (e.g., Chao1 indices of 92.8 vs. 87.6) and 22% greater β-glucosidase activity than annual-only sequences. The relative abundances of the two most abundant prokaryotic phyla - *Actinobacteriota* and *Proteobacteria* - as well as the second most abundant fungal class, *Dothideomycetes*, followed the same trend. The soils with more frequent recurrence of grassy perennials in the sequences exhibited greater MBC (34%), higher prokaryotic Shannon diversity, greater fungal richness, and higher arylsulfatase activity (68%) than soils with more frequent recurrence of perennial legumes, although the predominant prokaryotic phylum, *Actinobacteriota* was more abundant in legume-based systems. The cropping sequences dominated by creeping red fescue grass seed crops exhibited the greatest improvement in most of the soil microbial metrics studied. Nitrogen fertilizer increased the relative abundance of the copiotrophic *Actinobacteriota* but decreased that of the oligotrophic *Acidobacteriota*. Prokaryotes were associated with C, N, P and S cycling, whereas fungi were primarily linked to C cycling. Overall, diversifying annual grain cropping systems with perennial forage seed crops, particularly creeping red fescue, enhanced key indicators of biological soil health.

## 1 Introduction

To meet the increasing demands for food, feed, fiber and energy without compromising ecosystem sustainability, crop production systems must maintain or improve soil health. Soil health has been defined as the continued capacity of soil to function as a vital living ecosystem that sustains plants, animals and humans (Lehmann et al., 2020). This definition views soil as a dynamic system in which physical, chemical, and biological properties interact to support the delivery of ecosystem services (Fierer et al., 2021; Toor et al., 2021). Maintaining soil health is therefore foundational to ensuring long-term productivity, resilience, and sustainability of agroecosystems worldwide. Soil microbial communities and their functions in the crop rhizosphere play a vital role in maintaining soil health. They contribute through a range of services, including phytohormone production, nutrient provision via biological nitrogen fixation and nutrient cycling, enhanced tolerance to abiotic stress, and the induction of the plant immune responses (Bakker et al., 2012). Other biological processes that generate positive ecosystem outcomes include biological pest control through microbial predation and competition for resources (Jaiswal et al., 2022), and detoxification (biodegradation) of agrochemicals (Dhuldhaj et al., 2023). Because these biologically mediated processes underpin key ecosystem services, management practices that foster soil microbial communities are central to ecologically regenerative agriculture. One way to improve biological soil health and boost these biological processes is to increase soil organic C because it is the main substrate for heterotrophic soil microbial communities. We investigated whether adding perennial crops to annual crops could offer such benefits because perennial crops produce more biomass, possess deeper root systems, provide more continuous soil cover, and experience less disturbance, resulting in greater soil organic C inputs and reduced C losses than annual crops (Gentile et al., 2005; Ghimire et al., 2018). Identifying cropping system strategies that enhance soil organic C while sustaining farm profitability remains a critical challenge for agroecosystems management. In temperate regions of North America, Australia and Europe, wheat-canola-pulse rotations predominate (Kirkegaard et al., 2008), yet they remain sparsely diversified with other annual or perennial crops that could enhance systems resilience and profitability (Simão et al., 2024). Seed crops of cool season forages such as clovers, creeping red fescue, timothy and bromegrasses are included in the crop rotations in the Canadian Prairies, temperate USA (Steiner and Springer, 2020), New Zealand (Rolston et al., 2006), Denmark (Wong, 2021), and many other countries (Burgon et al., 1998). In the Peace River region located in northwestern Canada, perennial forage seed production has historically contributed substantially to the agriculture economy, with Canada emerging as a global leader in forage and turfgrass seed exports (Agriculture and Agri-Food Canada, 2020). Despite their economic importance and agroecological significance, the perennial forage and turfgrass seed crop acreage has declined as producers increasingly adopt simplified annual crop rotations (Khanal, 2023). Such simplification has contributed to increased vulnerability to crop pests and diseases (McCallum et al., 2021; Strelkov et al., 2021; Chin et al., 2023), herbicide resistant weeds (Beckie et al., 2020), and environmental stress, partly due to declining soil health. These trends represent not only an agronomic and environmental concern, but also a missed opportunity to leverage originally adapted perennial crops to enhance the resilience and sustainability of prairie cropping systems. Taken together, these trends highlight a widening gap between potential benefits of perennial forage seed crops and their declining presence in the prevailing crop rotations. Addressing this gap has direct implications for soil health improvement, crop system resilience, and the long-term viability of the forage seed sector. This disconnect raises a critical question: could integrating perennial forage seed crops into these annual grain systems simultaneously diversify rotations, improve soil health, and revitalize the forage seed sector?

Studies comparing annual and perennial systems have consistently reported greater soil microbial biomass, altered community composition, and enhanced nutrient transformation under perennial vegetation. Alagele et al. (2020) reported greater soil MBC and higher abundance of all microbial groups as determined by phospholipid fatty acid (PLFA) analysis, under perennial vegetation treatments, including grass buffers and biomass crops, compared to the conventional corn-soybean rotation. Findings reported by Udawatta et al. (2024) corroborate increases in soil microbial biomass, and shifts in microbial community structure after one year of Kernza (*Thinopyrum intermedium*) and alfalfa perennial cropping. Similarly, Tang et al. (2024) found higher soil MBC and respiration rates in perennial intermediate wheatgrass than in annual wheat systems. Chantigny et al. (1996) further elucidated that soil MBC, denitrification potential and nitrification capacity were greater under perennial crops, such as timothy, bromegrass and reed canary grass, than under annual crops including wheat and faba bean, across most soil sampling periods. Collectively, these findings suggest that the soil microbial effects of integrating perennial and annual crops are strongly influenced by crop species and environmental conditions, emphasizing their context-specificity. This context-specificity underscores the need of generating region-specific evidence for the Canadian prairies, where long-term comparisons remain scares.

This study seeks to address a critical research gap in understanding how integrating perennial seed crops into the annual grain crop sequences influences soil microbial communities on the Canadian prairies. Filling this knowledge gap is essential for informing science-based crop diversification strategies that align agronomic productivity with soil health goals in prairie agroecosystems. Therefore, the objective of this study was to address this knowledge gap by evaluating the effects of integrating perennial forage seed crops into annual grain and oilseed crops sequences on soil microbial communities and their functional attributes. We hypothesized that integrating perennial forage seed crops, particularly legumes, into annual crop sequences would enhance the biological soil health by increasing microbial biomass, taxonomic diversity and enzyme activities. Using a 10-year field experiment with eight distinct cropping sequences under two N fertilization rates and an unfertilized control, we examined soil MBC, the composition and diversity of prokaryotic (bacterial and archaeal) and fungal communities, and the activities of key enzymes involved in C, N, P and S cycling. By leveraging a long-term field experiment, this study provides critical insights into the lasting impacts of perennial crop integration on belowground biological processes.

## 2 Materials and methods

### 2.1 Study site, treatments, and crop management

The field experiment was established at the Beaverlodge Research Farm of Agriculture and Agri-Food Canada (55°12’ N 119°24’ W) near the town of Beaverlodge, Alberta, Canada, in 2013. The location lies in a continental boreal climate with average monthly temperatures ranging from 15 °C in the warmest month of July to -11 °C in the coldest month of January, and annual precipitation averages approximately 434 mm. The soil is a Dark Gray Luvisol in the Canadian soil classification system (Soil Classification Working Group, 1998), a Haplocryalf in the USDA taxonomy (Soil Survey Staff, 2022), and an Albic Luvisol in the World Reference Base for Soil Resources (IUSS Working Group WRB, 2022). The baseline soil properties in the 0–15 cm soil depth in 2013 were: pH 5.43, 3.23% organic C, 31.3% sand, 46.0% silt and 22.7% clay.

The experiment had a split-plot design with four replications. Treatments included eight main plots of different cropping sequences and three sub-plots of nitrogen rates of 0, 45 and 90 kg N ha^-1^ (N0, N45 and N90). The cropping sequences involved different combinations of four annual crops: barley (*Hordeum vulgare* L.), canola (*Brassica napus* L.), pea (*Pisum sativum* L.) and wheat (*Triticum aestivum* L.), and four perennial forage seed crops: alsike clover (*Trifolium hybridum* L.), creeping red fescue (*Festuca rubra* L. ssp. *rubra*), meadow bromegrass (*Bromus riparius* Rehmann) and red clover (*Trifolium pratense* L.). **Table 1** presents the functional characterization of the cropping sequences. Each cropping sequence contained three levels of nitrogen as sub-plots. The sub-plot size was 2.5 m wide and 8 m long, with eight rows spaced 30 cm apart. Crop management practices included no-till air-seeding, pre-seed and post-emergence herbicide applications, and applications of phosphorus (P_2_O_5_), potassium (K_2_O), and sulfur (S) at rates of 30, 20 and 20 kg ha^-1^, respectively. Fertilizers were band-applied in spring for annual crops and broadcast-applied in fall for perennial seed crops. Details of the crop management practices are described in a previous study (Khanal et al., 2021).

**Table 1:** Crop sequencies^a^, each at N fertilizer rates of 0, 45 and 90 kg N ha^-1^.

Designation	Crop sequence, 2013–2022	Key/transitioning features
S1	W-C-W-C-W-C-W-C-FR-C	Conventional annual crops
S2	P-B-W-C-P-W-C-P-FR-W	Diversified annual crops
S3	C-C-C-C-P-MB-MB-MB-FR- P	Annual to perennial crops
S4	RC-RC-W-C-RC/W-RC-W-C-FR-MB	Perennial legume to vernalizing grass crops
S5	AC-AC-W-C-AC/W-AC-W-C-FR-T	Perennial legume to non-vernalizing grass crops
S6	inRC-RC-W-C-RC/W-RC-C-W-FR-CF	Perennial legume to turf grass crops
S7	CF-CF-CF-C-P-CF-CF-CF-FR-P	Turf grass to perennial legume crops
S8	inAC-AC-W-C-AC/W-AC-CF-CF-FR-C	Perennial to annual crops

^a^ C, Canola; W, Wheat; B, Barley; P, Pea; MB, Meadow bromegrass; T, Timothy; CF, Creeping red fescue; RC, Red clover; AC, Alsike clover; FR, Fall rye grass; in, Inoculated with rhizobium. Fall rye (FR) was seeded in all plots in 2021 due to drought-induced crop failure.

### 2.2 Soil sampling

Ten years after the trial was established, we collected soil samples to a depth of 7.5 cm from four locations per plot on October 4 of 2022, after annual crop harvest. We combined the four soil samples from each plot into a composite sample and passed the composite through a 2 mm sieve. We froze the samples for DNA extraction at -20 °C, stored samples designated for enzyme analysis at 4 °C, and air-dried samples designated for permanganate-oxidizable C (POXC) and microbial biomass C (MBC) analysis before storing them at 4 °C.

### 2.3 Permanganate-oxidizable C and microbial biomass C

We extracted POXC (active or labile C) with 0.02 M KMnO_4_ and read its absorbance with a spectrophotometer at a wavelength of 550 nm (Soil Survey Staf, 2022). We used the substrate-induced respiration method (Horwath and Paul, 1994) to measure soil MBC. We dissolved 300 mg of glucose in 9.0 mL water and added it to 50 g of air-dry soil to bring it to 50% of water-holding capacity (determined by measuring the gravimetric water content after draining a water-saturated column of soil until the water stopped dripping). After stir-mixing, we incubated the soil in a 1-L jar for 3 h at 22 °C and measured the amount of CO_2_ that accumulated in the head space using gas chromatography.

### 2.4 DNA extraction and sequencing

We extracted soil DNA with the Qiagen DNeasy PowerLyzer PowerSoil kit (Qiagen, Toronto, Ontario) according to the manufacturer’s instructions. MiSeq DNA sequencing was done at the Centre for Health Genomics and Informatics of the University of Calgary, following the Qiagen 16S v4/v5 region and fungal internal transcribed spacer (ITS) region library preparation. The 16S amplicon forward primer was 5’ TCGTCGGCAGCGTCAGATGTGTATAAGAGACAGCCTACGGGNGGCWGCAG, and the reverse primer was 5’ GTCTCGTGGGCTCGGAGATGTGTATAAGAGACAGGACTACHVGGGTATCTAATCC (Klindworth et al., 2013). For fungi, the forward ITS1 primer was 5’ CTTGGTCATTTAGAGGAAGTAA, the reverse ITS2 primer was 5’ GCTGCGTTCTTCATCGATGC) (Bellemain et al., 2010).

### 2.5 Bioinformatics analysis

Using QIIME2 v2024.05 (Bolyen et al., 2019), we demultiplexed and denoised the 16S sequences with Divisive Amplicon Denoising Algorithm, DADA2 (Callahan et al., 2016). We assigned taxonomy with a Naïve Bayes classifier trained for the 16S rRNA gene target region against the SILVA 138, 99% operational taxonomic unit (Lofgren et al.), reference database (Quast et al., 2013). For the ITS sequence analysis, we used the PIPITS 3.0 pipeline (GitHub - hsgweon/pipits: Automated pipeline for analyses of fungal ITS from the Illumina) to produce amplicon sequence variant (ASV) abundance and taxonomic assignment tables, after taxonomic assignment of sequences using Ribosomal Database Project (RDP) Classifier against the UNITE fungal ITS reference dataset (Gweon et al., 2015). For both 16S and ITS, we exported the OTUs/ASVs and taxonomic assignment tables to the Marker-gene Data Profiling (MDP) module of the online platform MicrobiomeAnalyst for further bioinformatics analysis (https://www.microbiomeanalyst.ca/) (Chong et al., 2020; Lu et al., 2023). We filtered the sequences for low counts and low variance before analysis. We calculated the relative microbial abundances at various classification levels from phylum to genus. We calculated the following α-diversity indices at OTU/ASV level: ACE (abundance-based coverage estimator), Chao1, Fisher and Shannon, after rarefying the data to the minimum library size. We visualized β-diversity using Principal Coordinate Analysis (PCoA) on a Bray-Curtis dissimilarity index and assessed group differences in microbial community structures for statistical significance using permutational multivariate analysis of variance (PERMANOVA), all in MicrobiomeAnalyst. To identify differentially abundant taxa between treatments, we used the Linear discriminant analysis Effect Size (LEfSe) method within MicrobiomeAnalyst.

### 2.6 Enzyme activities

We measured the activities of the extracellular enzymes β*-*glucosidase (C cycling), N*-*acetyl*-*β-glucosaminidase (NAG) (N cycling) and acid phosphomonoesterase (P cycling) using microplate fluorimetric assays (Deng et al., 2011) as described by Lupwayi et al. (2019). These assays were based on the detection of 4-methylumbelliferone (MUF) released by the enzymatic hydrolysis of MUF-labeled substrates incubated with soil at the optimal pH of each enzyme. We quantified the arylsulfatase activity in a bench-scale assay through colorimetric determination of the *p*-nitrophenol released by the enzyme after incubating 1 g of soil with buffered (pH 6.0) *p*-nitrophenyl-β-*D*- sulfate (Dick et al., 1997).

### 2.7 Statistical analysis

We used analysis of variance (ANOVA) in Statistix software version 9 (Analytical Software, FL, USA) to statistically evaluate the effects of crop sequences and nitrogen rates on the soil POXC, MBC, relative abundances of the microbial communities at selected classification levels, α-diversity indices, and enzyme activities. The experimental design was split plot, with crop sequences as main plots and nitrogen rates as sub-plots. Main plot (crop sequences, S), sub-plot (nitrogen rates, N) and their interactions (S*N) were specified as fixed factors, and the replicates (R) and S*R as random factors in the analytical model. We used the 5% level of significance to determine statistical significance, and separated means using the Tukey HSD test when effects were significant. To further examine the effects of crop sequences, we constructed the following orthogonal contrasts, as described by Vargas et al. (2018):

- Annual crops only vs. succession of perennial and annual crops: Sequences S1 and S2 vs. Sequences S3-S8 (**Table 1**).
- Perennial legumes dominant vs. Perennial grasses dominant: Sequences S4-S6 and S8 vs. Sequences S3 and S7 (**Table 1**).

We used Pearson correlation analysis to assess the relationships between enzyme activities and the relative abundances of the most abundant genera, prokaryotic phyla, and fungal classes.

## 3 Results

### 3.1 POXC and MBC

Irrespective of N rate, crop sequence S8 had the highest soil POXC content, although only sequences S3 and S5 had significantly lower POXC content (**Table 2**). Contrast analysis did not reveal any differences in soil POXC contents between crop sequences with a succession of perennial and annual crops and those with annual crops only, nor between crop sequences predominated by grass perennials and those predominated by legume perennials. The N45 nitrogen rate decreased POXC relative to the N0 rate, but the N90 rate increased it relative to both the N0 and N45 rates; thus, the N45 rate had the lowest POXC content and N90 the highest.

**Table 2:** Soil carbon fractions in the different crop sequences and nitrogen treatments.

Treatment	Permanganate-oxidizable C (POXC)	Microbial biomass C (MBC)
	(mg C kg^-1^ soil)	
Crop sequence (S)		
S1	665 ab^a^	664 bc
S2	682 ab	718 bc
S3	587 b	930 ab
S4	661 ab	592 c
S5	591 b	692 bc
S6	723 ab	636 c
S7	682 ab	1023 a
S8	766 a	986 a
SEM^b^	31.0	56.7
Nitrogen (N, kg ha^-1^)		
0	679 b	798 a
45	603 c	821 a
90	726 a	722 a
SEM		46.8
S x N	NS^c^	*^d^
Contrasts (Significant contrast means are in brackets)		
Annual vs. Succession	NS	Succession (810) > Annual (691)^e^
Legumes vs. Grasses	NS	Grasses (976) > Legumes (726)

^a^ Means followed by the same letter are not significantly different at 5% significance level.

^b^ SEM, Standard error of the means.

^c^ NS, Not significant at 5% significance level.

^d^ *Significant at 5% significance level.

^e^ The values in parenthesis are the means for the treatments in the contrasts (listed in Materials and Methods). See “Statistical analysis” in Materials and Methods for the treatments that comprise each contrast.

The response of soil MBC contents to crop sequences depended on N rates, i.e., there was a significant interaction between the two factors (**Table 2**). Crop sequences S7 and S8 had the highest soil MBC, with MBC in sequence S8 being enhanced at the N90 rate. The N90-induced increase also occurred in crop sequence S3, but this N rate reduced MBC relative to N0 in the remaining crop sequences. Contrast analysis showed that the crop sequences with a succession of perennial and annual crops had 17% greater soil MBC (810 mg C kg^-1^ soil) than sequences with annual crops only (691 mg C kg^-1^ soil), and that the crop sequences predominated by grass perennials had 34% greater MBC than those predominated by legume perennials (976 vs. 726 mg C kg^-1^ soil).

### 3.2 Prokaryotic communities

**Figure 1:**
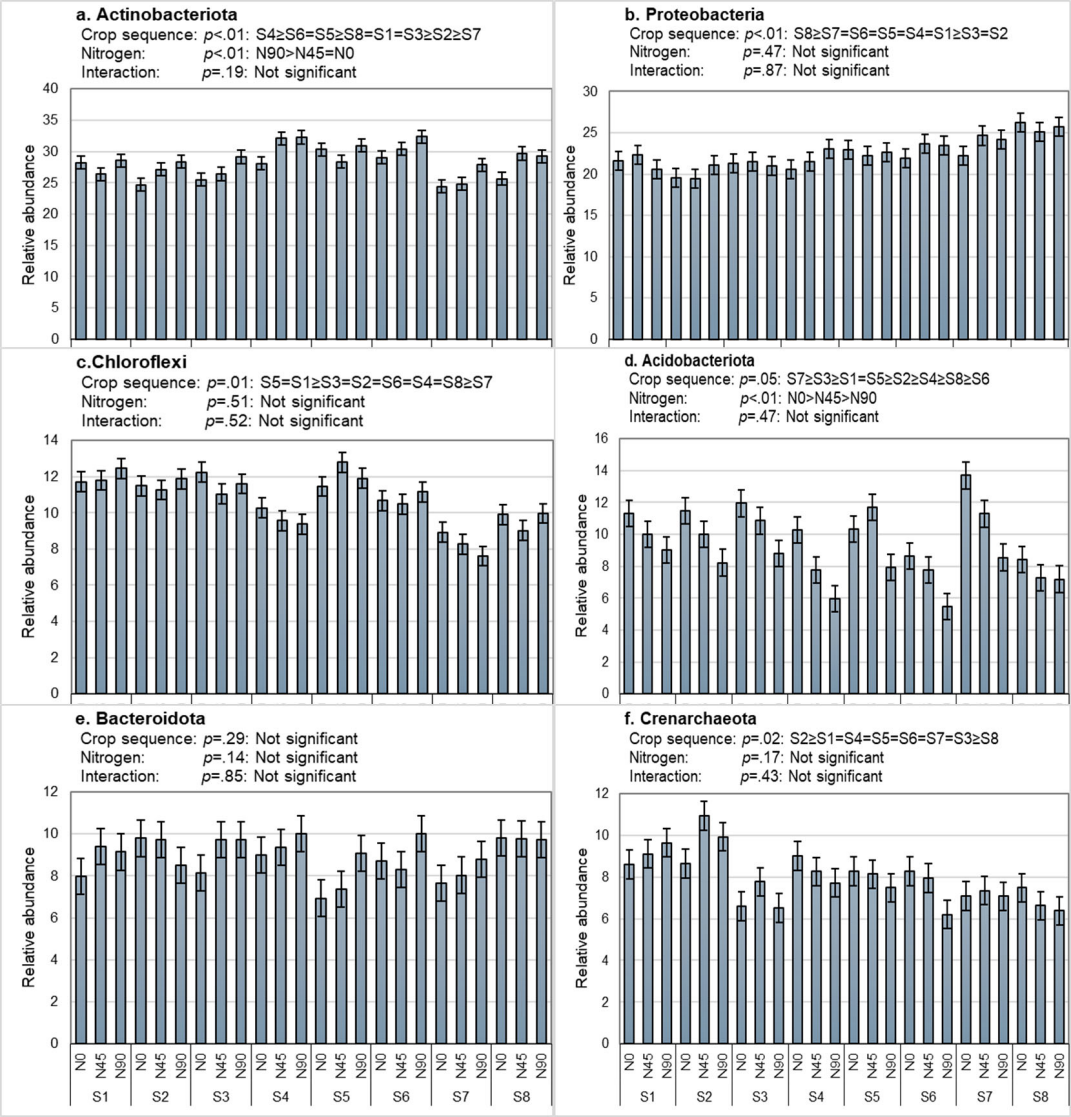
Crop sequence and nitrogen effects on the relative abundances of the six most abundant prokaryotic phyla (in descending order): *Actinobacteriota* **(A)**, *Proteobacteria* **(B)**, *Chloroflexi* **(C)**, *Acidobacteriota* **(D)**, *Bacteriodota* **(E)** and *Crenarchaeota* **(F)**. The error bars represent the standard error of the mean (SEM).

There were no interactions between crop sequence and nitrogen rate in the relative abundances of prokaryotic phyla (**Figure 1**; **Supplementary Table 1**). The six most abundant phyla responded differently to crop sequences: whereas crop sequences S7 and/or S8 had the highest relative abundances of *Proteobacteria* (**Figure 1B**) and *Acidobacteriota* (**Figure 1D**), at least one of them had the lowest relative abundances of *Actinobacteriota* (**Figure 1A**), *Chloroflexi* (**Figure 1C**) and *Crenarchaeota* (**Figure 1F**). Nonetheless, contrast analysis revealed that the two most abundant phyla (*Actinobacteriota* and *Proteobacteria*) were more abundant in crop sequences with a succession of perennial and annual crops than in those with only annual crops, but the reverse was observed for *Chloroflexi* and *Crenarchaeota*. Among the perennial sequences, those predominated by legume perennials had a higher relative abundance of *Actinobacteriota* than those predominated by grass perennials, but the reverse was true for *Acidobacteriota.* The relative abundances of *Actinobacteriota* increased with increasing rate of N (**Figure 1A**), but the reverse was true for *Acidobacteriota* (**Figure 1D**).

**Figure 2:**
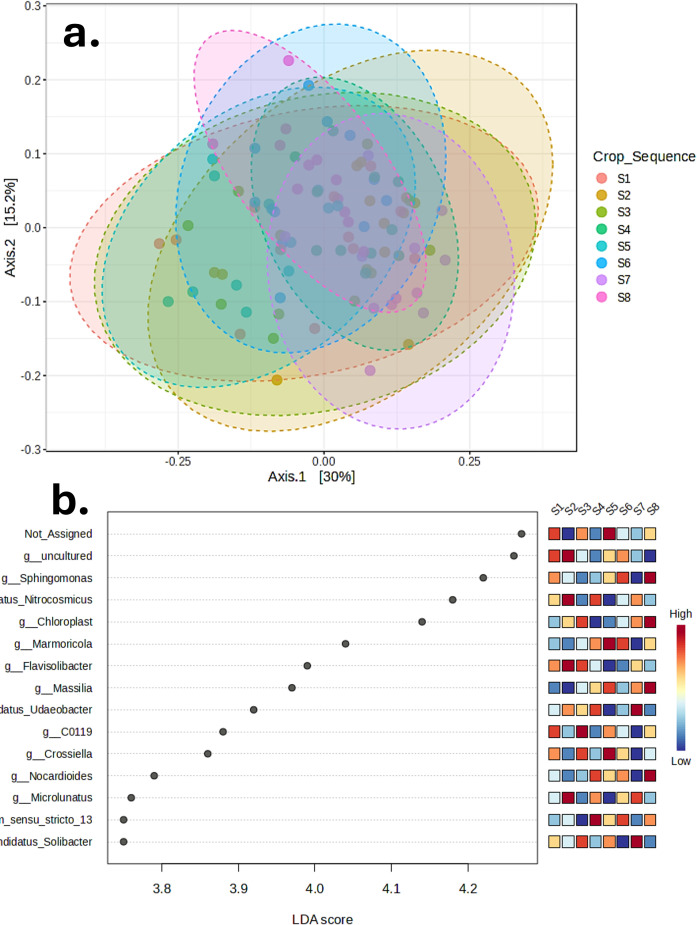
Prokaryotic community structures (β-diversity) in the different crop sequences **(A)** as determined by Principal Coordinate Analysis (PCoA) (PERMANOVA F-value = 3.998, r^2^ = 0.243, p = 0.001), and the differential abundances of the prokaryotic genera in the different crop sequences as determined by LEfSe **(B)**. In **(B)**, the names cut off on the y-axis are g_Candidatus_Nitrocosmicus, g_Candidatus_Udaeobacter, g_Candidatus_Solibacter, and g_Clostridium_sensu_stricto_13.

**Figure 3:**
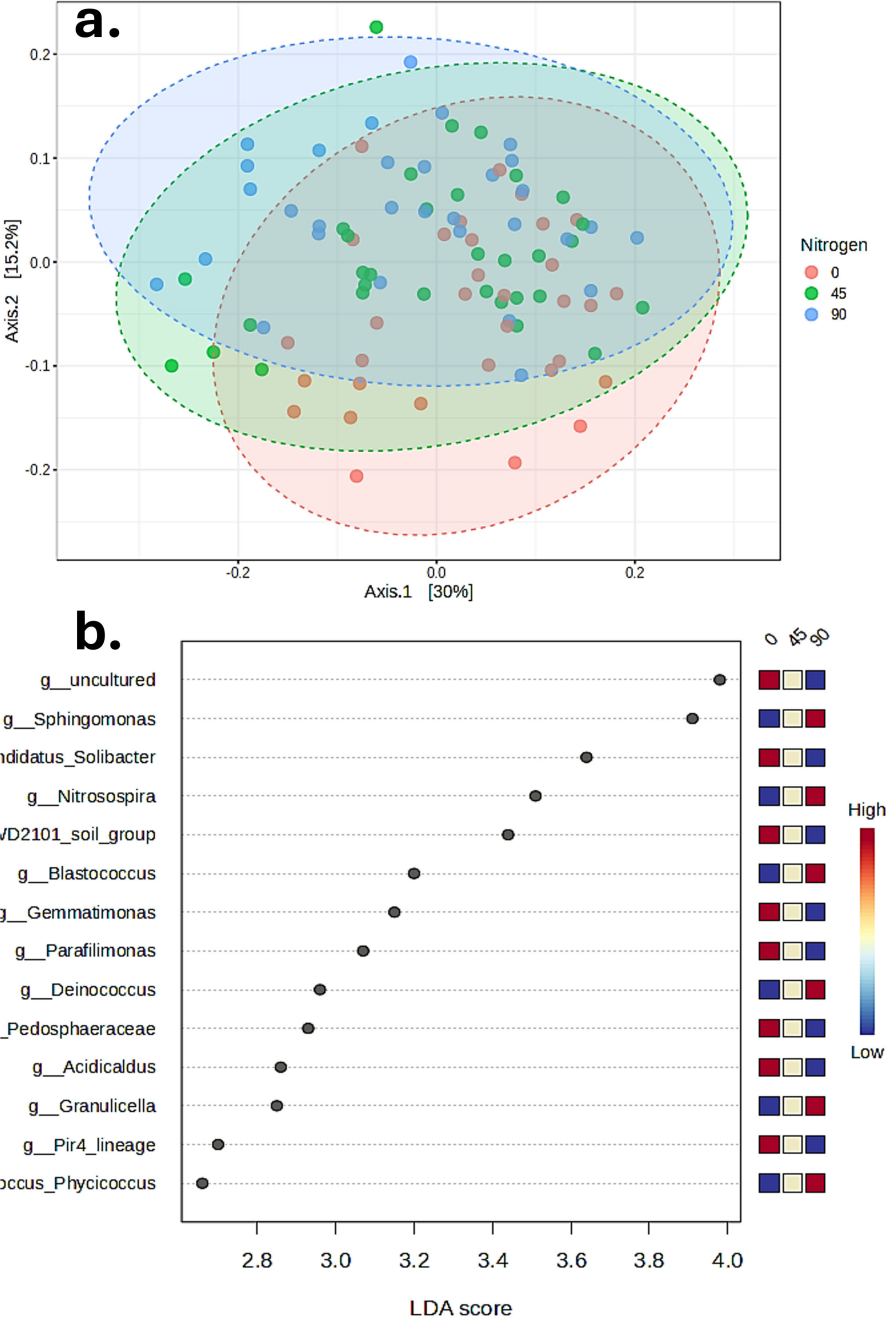
Prokaryotic community structures (β-diversity) in the different nitrogen treatments **(A)** as determined by Principal Coordinate Analysis (PCoA) (PERMANOVA F-value = 2.891, r^2^ = 0.059, p = 0.002), and the differential abundances of the prokaryotic genera in the different nitrogen treatments as determined by LEfSe **(B)**. In **(B)**, the names cut off on the y-axis are g_Candidatus_Solibacter, g_WD2101_soil_group, g_Pedosphaeraceae, and g_Pedococcus_Phycicoccus.

The above differences in the relative abundances of prokaryotic phyla are reflected in the differences in β-diversity (community structures) among crop sequence (PERMANOVA F-value = 3.998, r^2^ = 0.243, p = 0.001) and nitrogen (PERMANOVA F-value = 2.891, *r^2^* = 0.059, *p* = 0.002) treatments. Principal Coordinate Analysis (PCoA) revealed that crop sequences S7 exhibited a distinct prokaryotic community structure compared with the other crop sequences (**Figures 2A, B**; **Supplementary Table 2**). This crop sequence had the lowest relative abundances of the three most abundant genera: *Sphingomonas* (phylum *Proteobacteria*)*, Marmaricola* (phylum *Actinomycetota*) and *Nocardioides* (phylum *Actinomycetota*). PCoA also revealed that the prokaryotic community structure in N0 treatment differed from those receiving nitrogen fertilization (N45 and N90) (**Figure 3A**). Although LEfSe showed that only *Sphingomonas* among these three genera was least abundant at N0 (**Figure 3B**), ANOVA showed that all three genera were least abundant in the crop sequence S7 (**Supplementary Table 2**).

The richness indices of α-diversity (ACE, Chao1 and Fisher) were not affected by crop sequence or nitrogen (**Table 3**). However, crop sequence S7 had significantly greater Shannon index than the other sequences except S8, and nitrogen decreased this index (*p* < 0.05) (**Table 3**). Contrast analysis showed that Shannon index was greater in the crop sequences that were dominated by grass perennials (4.20) than in those that were dominated by legume perennials (4.10).

**Table 3:** Prokaryotic α-diversity in the different crop sequences and nitrogen treatments.

Treatment	Richness index			Diversity index
	ACE	Chao1	Fisher	Shannon
Crop sequence (S)				
S1	105.8 a^a^	106.0 a	21.3 a	4.07 b
S2	100.2 a	100.3 a	19.9 a	4.08 b
S3	107.8 a	108.0 a	21.8 a	4.15 b
S4	105.6 a	105.3 a	21.2 a	4.13 b
S5	105.5 a	105.7 a	21.3 a	4.08 b
S6	106.6 a	106.7 a	21.5 a	4.08 b
S7	113.7 a	113.7 a	23.3 a	4.24 a
S8	112.1 a	111.9 a	22.9 a	4.12 ab
SEM^b^	3.80	3.80	0.92	0.035
Nitrogen (N, kg ha^-1^)				
0	109.2 a	109.3 a	22.2 a	4.16 a
45	106.2 a	106.2 a	21.4 a	4.10 b
90	106.0 a	106.1 a	21.4 a	4.09 b
SEM	2.00	1.99	0.49	0.016
S x N	NS^c^	NS	NS	NS
Contrasts (Significant contrast means are in brackets)				
Annual vs. Succession	N/A^d^	N/A	N/A	NS
Legumes vs. Grasses	N/A	N/A	N/A	Grasses (4.20) > Legumes (4.10)^e^

^a^ Means followed by the same letter are not significantly different at 5% significance level.

^b^ SEM, Standard error of the means.

^c^ NS, Not significant at 5% significance level.

^d^ N/A, Not applicable because crop sequences had no effect.

^e^ The values in parenthesis are the means for the treatments in the contrasts (listed in Materials and Methods). ACE (abundance-based coverage estimator), Chao1 and Fisher indices are richness indices, and Shannon index is an overall diversity index, i.e., a function of richness and evenness. See “Statistical analysis” in Materials and Methods for the treatments that comprise each contrast.

### 3.3 Fungal communities

**Figure 4:**
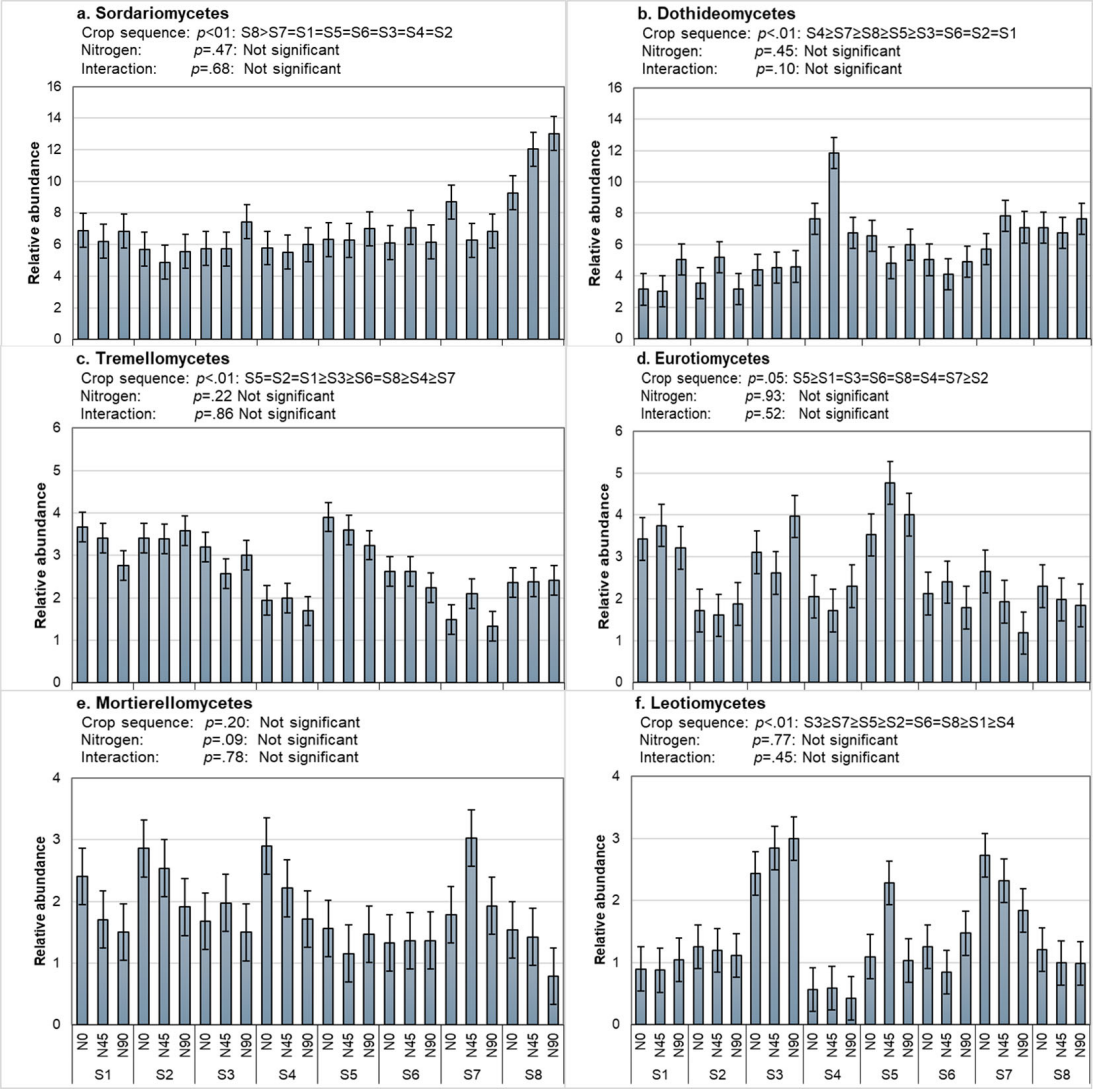
Crop sequence and nitrogen effects on the relative abundances of the six most abundant fungal classes (in descending order): *Sordariomycetes* **(A)**, *Dothideomycetes* **(B)**, *Tremellomycetes* **(C)**, *Eurotiomycetes* **(D)**, *Mortierellomycetes* **(E)** and *Leotiomycetes* **(F)**. The error bars represent the standard error of the mean (SEM).

Fungal community composition varied across crop sequences, with two most abundant fungal classes showing sequence-specific patterns (**Figure 4**; **Supplementary Table 3**). *Sordariomycetes* were most abundant in crop sequence S8 (**Figure 4A**), whereas *Dothideomycetes* were most abundant in crop sequence S4, particularly at N45 rate (**Figure 4B**). According to contrast analysis, *Dothideomycetes* were more abundant in crop sequences with a succession of perennial and annual crops than in those with annual crops only. By contrast, crop sequence S4 had the lowest relative abundance of *Leotiomycetes* (**Figure 4F**) and the second lowest relative abundance of *Tremellomycetes* (**Figure 4C**). Whereas *Leotiomycetes* were more abundant in crop sequences with a succession of perennial and annual crops than in those with annual crops only, according to contrast analysis, the reverse was true for *Tremellomycetes*. Within the crop sequences containing perennial crops, those sequences predominated by grasses had a higher relative abundance of *Leotiomycetes* than those predominated by legumes. Except for the interactive effect of nitrogen with crop sequence on the relative abundance of *Dothideomycetes* (**Figure 4B**), nitrogen had no effects on the relative abundances of fungal classes.

**Figure 5:**
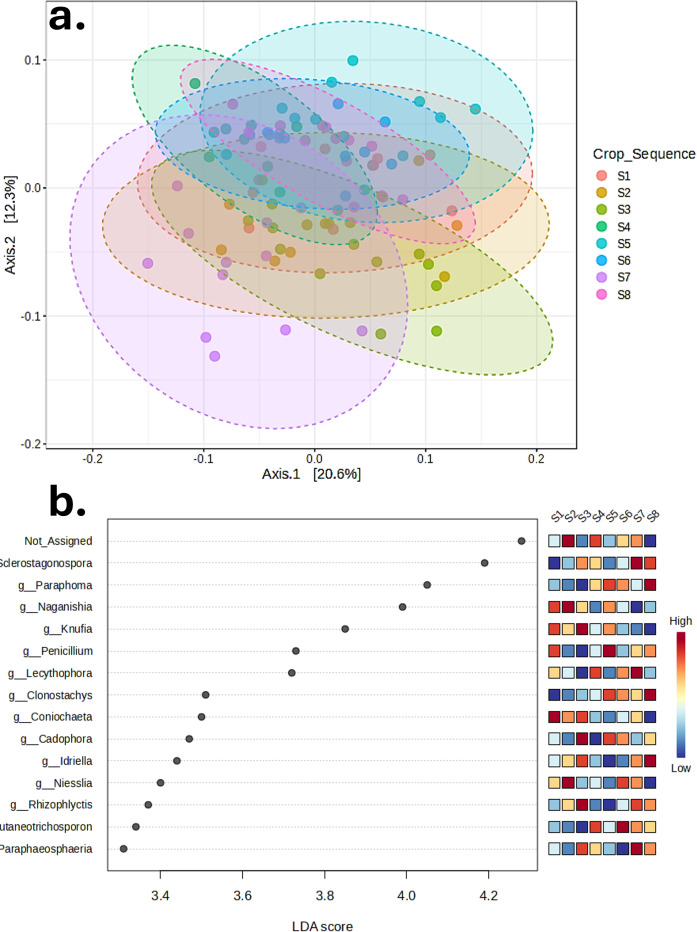
Fungal community structures (β-diversity) in the different crop sequences **(A)** as determined by principal coordinate analysis (PCoA) (PERMANOVA F-value = 7.285, r^2^ = 0.370, p = 0.001), and the differential abundances of the prokaryotic genera in the different crop sequences as determined by LEfSe **(B)**.

Analysis of β-diversity by PCoA (PERMANOVA F-value = 7.285, *r^2^* = 0.370, *p* = 0.001) showed that crop sequences S8, S3 and S7 exhibited distinct fungal community structures compared with the other crop sequences (**Figure 5A**). Of the four most abundant fungal phyla, both LEfSe (**Figure 5B**) and ANOVA (**Supplementary Table 4**) consistently showed that the genera *Naganishia* (class *Tremellomycetes*) was most abundant in crop sequence S2 and least abundant in sequence S7, whereas *Sclerostagonospora* (class *Dothideomycetes*) and *Paraphoma* (class *Dothideomycetes*) were most abundant in crop sequences S7 or S8 and least abundant in S1 or S3. Nitrogen had no effect on β-diversity.

The richness indices of the soil fungal α-diversity were highest in crop sequences S8 and lowest in sequence S2, but the Shannon index was not affected (**Table 4**). According to contrast analysis, the richness indices were greater in the crop sequences with a succession of perennial and annual crops than in those with only annual crops (e.g., Chao1 indices of 92.8 vs. 87.6), and were also greater in crop sequences that were predominated by grass perennials than in those predominated by legume perennials (e.g., Chao1 indices of 96.1 vs. 91). Nitrogen has no effect on any of these indices.

**Table 4:** Fungal α-diversity in the different crop sequences and nitrogen treatments.

Treatment	Richness index			Diversity index
	ACE	Chao1	Fisher	Shannon
Crop sequence (S)				
S1	89.0 bc^a^	90.6 abc	13.5 bc	2.11 a
S2	84.9 c	84.6 c	12.8 c	2.04 a
S3	94.5 ab	95.0 abc	14.9 ab	2.25 a
S4	89.9 abc	90.5 abc	14.0 abc	2.18 a
S5	87.6 bc	87.3 bc	13.6 bc	2.24 a
S6	87.4 bc	86.9 bc	13.5 bc	2.15 a
S7	95.2 ab	97.2 ab	15.1 a	2.22 a
S8	97.4 a	99.8 a	15.3 a	2.30 a
SEM^b^	1.68	2.30	0.30	0.055
Nitrogen (N, kg ha^-1^)				
0	89.1 a	90.6 a	13.8 a	2.18 a
45	92.0 a	92.7 a	14.3 a	2.20 a
90	91.1 a	91.1 a	14.2 a	2.17 a
SEM	1.14	1.43	0.18	0.019
S x N	NS^c^	NS	NS	NS
Contrasts (Significant contrast means are in brackets)				
Annual vs. Succession	Succession (92.0) > Annual (86.9)^d^	Succession (92.8) > Annual (87.6)	Succession (14.4) > Annual (13.1)	N/A^e^
Legumes vs. Grasses	Grasses (94.9) > Legumes (90.6)	Grasses (96.1) > Legumes (91.1)	Grasses (15.0) > Legumes (14.1)	N/A

^a^ Means followed by the same letter are not significantly different at 5% significance level.

^b^ SEM, Standard error of the means.

^c^ NS, Not significant at 5% significance level.

^d^ The values in parenthesis are the means for the treatments in the contrasts (listed in Materials and Methods).

^e^ N/A, Not applicable because crop sequences had no effect. ACE (abundance-based coverage estimator), Chao1 and Fisher indices are richness indices, and Shannon index is an overall diversity index, i.e., a function of richness and evenness. See “Statistical Analysis” in Materials and Methods for the treatments that comprise each contrast.

### 3.4 Enzyme activities

**Figure 6:**
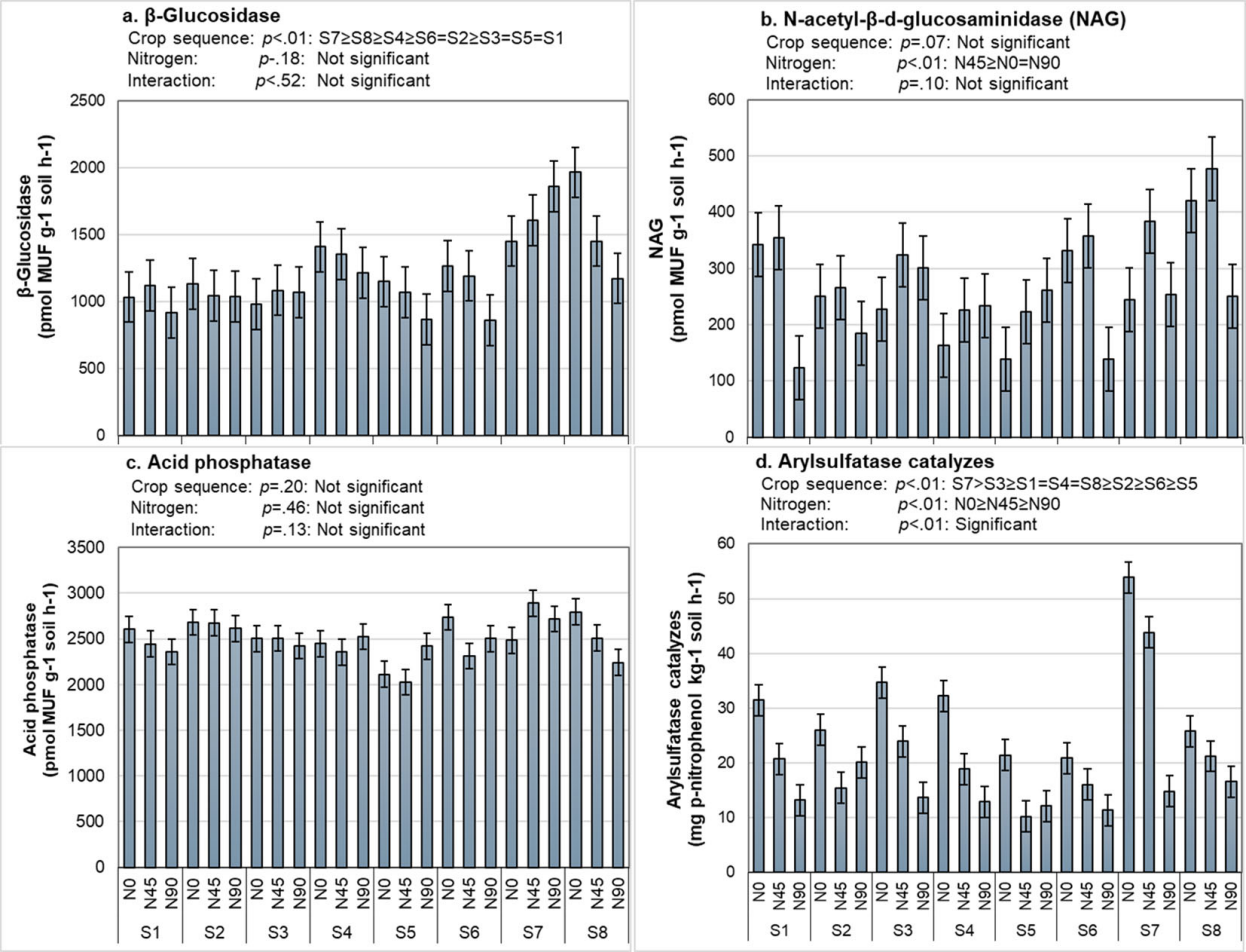
Crop sequence and nitrogen effects on the activities of soil extracellular enzymes: β*-*glucosidase **(A)**, N*-*acetyl*-*β-glucosaminidase **(B)**, acid phosphomonoesterase **(C)** and arylsulfatase **(D)**.

The activity of β-glucosidase was highest in crop sequence S7 regardless of nitrogen rate, which had no effect, and lowest in crop sequence S1 (**Figure 6A**). Contrast analysis revealed that the activity of this enzyme was 22% greater in crop sequences that included a succession of perennial and annual crops (1281 pmol MUF g^-1^ soil h^-1^) than those that included only annual crops (1049 pmol MUF g^-1^ soil h^-1^). Crop sequence did not affect the activity of N-acetyl-β-glucosaminidase, which was highest at N45 (**Figure 6B**). Acid phosphomonoesterase was unaffected by either crop sequence or nitrogen (**Figure 6C**). Arylsulfatase activity was highest in crop sequence S7 and decreased with increasing N rate in all crop sequences except in S2 and S5 where the N90 rate did not reduce the activity further after the reduction at N45 (**Figure 6D**). The crop sequences that were predominated by perennial grasses had 68% greater arylsulfatase activity (30.8 kg p-nitrophenol kg^-1^ soil h^-1^) than those that were predominated by legume perennials (18.3 kg p-nitrophenol kg^-1^ soil h^-1^).

### 3.5 Correlations between composition and function

Whereas the relative abundances of the four most abundant prokaryotic genera were either not correlated with, or negatively correlated with, the activity of β-glucosidase, the relative abundances of the next two most abundant genera were both positively correlated with β-glucosidase activity (**Table 5**). All the three observed significant correlations between the relative abundances of the six most abundant prokaryotic genera and N-acetyl-β-glucosaminidase activity were positive. By contrast, arylsulfatase activity was negatively correlated with the relative abundances of four of the six most abundant genera and positively correlated with only one. Acid phosphomonoesterase activity was correlated with the relative abundances of three prokaryotic genera, two of which exhibited positive correlations.

**Table 5:** Correlations (n = 95) of the relative abundances of the six most abundant prokaryotic and fungal genera (in descending order) with enzyme activities.

Genus (phylum for prokaryotes or Class for fungi)	Correlation coefficient (probability)			
	β-glucosidase	NAG	Acid phosph	Arylsulf
Prokaryotes				
*Sphingomonas* (*Proteobacteria*)				-0.446 (<0.001)
*Marmoricola* (*Actinomycetota*)	-0.331 (0.001)		-0.427 (<0.001)	-0.500 (<0.001)
*Nocardioides* (*Actinomycetota*)		0.210 (0.041)		-0.384 (<0.001)
*C01*19 (*Chloroflexi*)	-0.343 (<0.001)			
*Flavisolibacter* (*Bactereidota*)	0.309 (0.002)	0.242 (0.018)	0.453 (<0.001)	0.234 (0.022)
*Chloroplast* (*Cyanobacteria*)	0.239 (0.020)	0.238 (0.021)	0.263 (0.010)	
Fungi				
*Naganishia* (*Tremellomycetes*)	-0.413 (<0.001)			
*Knufia* (*Eurotiomycetes*)	-0.362 (<0.001)		-0.345 (<0.001)	
*Sclerostagonospora* (*Dothideomycetes*)	0.320 (0.002)			0.265 (0.009)
*Paraphoma* (*Dothideomycetes*)	0.234 (0.023)	0.261 (0.011)		
*Penicillium* (*Eurotiomycetes*)				
*Lecythophora* (*Sordariomycetes*)	0.235 (0.022)			0.344 (<0.001)

Only significant correlations are listed. NAG, N*-*acetyl*-*β-glucosaminidase; Acid phosph, acid phosphomonoesterase; Arylsulf, arylsulfatase.

Of the six most abundant fungal genera, only the relative abundances of the genera *Paraphoma* (which showed positive correlation) and *Knufia* (which showed negative correlation) were correlated with N-acetyl-β-glucosaminidase and acid phosphomonoesterase activities, respectively (**Table 5**). The relative abundances of the two most abundant fungal genera were negatively correlated with the activity of β-glucosidase.

## 4 Discussion

### 4.1 Annual crops only versus perennial and annual crops in sequence

The results of this study indicate that the inclusion of perennial forage crops into annual cropping sequences led to measurable improvements in biological soil health indicators. Soils in crop sequences that included perennial and annual crops in the succession had greater MBC (**Table 2**), higher fungal richness (**Table 4**), and increased β-glucosidase activity (**Figure 6A**) than soils in sequences composed solely of annual crops. The relative abundances of the two most abundant prokaryotic phyla, *Actinobacteriota* (**Figure 1A**) and *Proteobacteria* (**Figure 1B**), as well as the second most abundant fungal class, *Dothideomycetes* (**Figure 4B**), followed the same trend. LEfSe analysis also revealed similar differences in relative abundances in different crop sequences at genus level (**Figures 2B**, **5B**).

In terms of life strategy, the two prokaryotic phyla, *Actinobacteriota* and *Proteobacteria*, are generally classified as copiotrophic, i.e., most of their members proliferate in C- and nutrient-rich environments, in contrast to oligotrophs, which are adapted to grow in C- and nutrient-poor environments (Fierer et al., 2007, 2012). The contrasting responses of copiotrophs and oligotrophs across crop sequences provide further evidence that the inclusion of perennials improved soil biological health by increasing soil organic C. Consistent with this interpretation, soil POXC was positively correlated with the relative abundances of the prokaryotic phyla *Proteobacteria* and *Bacteroidota*, but negatively correlated with those of the oligotrophic phyla *Chloroflexi, Acidobacteriota*, and *Planctomycetota* (**Supplementary Table 5**). Similar findings have been reported in other studies, which demonstrated that perennial crops increase soil MBC (Udawatta et al., 2024), microbial α-diversity and the activity of N-acetyl-β-glucosaminidase (Erhunmwunse et al., 2023).

In all cases where the perennial crops enhanced the soil microbial properties described above, no differences were observed between the two crop sequences composed exclusively of annual crops (S1 and S2). Although the crop sequence S2 was more diverse, including pea, barley, wheat, and canola compared with sequence S1 which consisted only of wheat and canola, soil microbial properties did not differ between the two sequences. These results indicate that crop diversification through the inclusion of perennial crops has a more substantial beneficial impact on soil microbial communities in the surface soil layer than diversification achieved solely within annual cropping systems. The soil in this study was sampled to 7.5 cm depth in all plots due to resource limitations. Considering that perennial crops have deeper root systems than annual crops, sampling deeper would likely have revealed even more pronounced differences in the soil microbial communities between annual and perennial crops. For example, Taylor et al. (2023) reported differences in fungal community Shannon diversity or richness between perennial intermediate wheatgrass and annual wheat only in the deepest (60–90 cm) soil layer examined. However, difference between annual and perennial cropping systems are not restricted to rooting depth alone. Grasses, legumes and crucifers also differ substantially in root architectures (Marshall et al., 2016; Thiagarajan et al., 2018) and exudate chemistry (Seitz et al., 2023), which in turn influence nutrient acquisition, microbial symbiosis, soil structure, and soil porosity (Bodner et al., 2021; Wen et al., 2022).

### 4.2 Perennial grasses vs. perennial legumes

Within the crop sequences that included both perennial and annual crops in the succession, soil in sequences predominated by grass perennials showed higher MBC (**Table 2**), prokaryotic Shannon diversity (**Table 3**), and fungal richness (**Table 4**), and greater arylsulfatase activity (**Figure 6D**) than soils in sequences predominated by legume perennials. However, the reverse was observed for the relative abundance of the most predominant prokaryotic phylum, *Actinobacteriota* (**Figure 1A**). A major distinction between legumes and grasses is the ability of legumes to fix atmospheric nitrogen, and this additional nitrogen supply may be particularly important when the non-legumes in the study do not receive supplemental nitrogen. For example, Ma et al. (2023) reported that the decomposition of a leguminous forage increased soil organic C, total N and the abundance of genes associated with sub-metabolic pathway involved in C cycling more than the decomposition of a grass forage. Although grasses can also fix atmospheric N through free living bacteria like *Azospirillum* (Smercina et al., 2019), the quantity of N fixed is generally much lower than that fixed by legumes. In addition, a smaller fraction of the N fixed in the grass rhizosphere is taken up by the host plant, relative to N fixed in the nodules of legume hosts, because rhizosphere-fixed N is more susceptible to leaching losses. In our study, applied N was an experimental factor, and its addition increased the relative abundance of *Actinobacteriota* even in the legume-dominated crop sequences S4 and S6 (**Figure 1A**).

Another difference is that grasses have a higher root density, which supports soil aggregate stability (Arshad et al., 2004; Garcia et al., 2019) and thereby increases soil organic C by protecting it from erosion and decomposition (Arshad et al., 2011; Ali et al., 2023). This characteristic likely underlies the greater MBC, prokaryotic Shannon diversity, and fungal richness and arylsulfatase activity in soils from sequences predominated by grass perennials compared with those predominated by legume perennials.

Of the crop sequences that were predominated by grass perennials, S7 and, in some cases, S8 consistently showed the most favorable effects on the soil microbial metrics in this study. Crop sequence S7 was predominated by creeping red fescue, whereas S8 included two consecutive years of creeping red fescue in the last four years of the study (**Table 1**). Thus, creeping red fescue was likely a key driver of these observed effects. Creeping red fescue is known to produce the weed-suppressive *meta*-tyrosine in its root exudates (Zer et al., 2020); however, the effects of this compound on soil microbial communities and soil properties remain poorly understood. In the same region as the present study, Arshad et al. (2004) reported greater soil organic matter and soil aggregation (mean-weight diameter) under perennial red fescue or bromegrass than under an annual wheat-based crop rotation. Similarly, in a study conducted in China, red fescue grown alone or in mixture with Kentucky blue grass increased soil organic matter, bacterial and fungal diversity, and activities of urease, alkaline phosphomonoesterase, invertase and catalase enzymes compared with Kentucky blue grass grown alone (Xie et al., 2020). Together, these findings indicate that red fescue plays a key role in enhancing soil biological activity and nutrient cycling, thereby contributing to improved soil health in fescue-based systems.

### 4.3 Applied nitrogen effects

Nitrogen affected prokaryotes to a greater extent than fungi. Consistent with the life strategy classification of the prokaryotic phylum *Actinobacteriota* as copiotrophic and *Acidobacteriota* as oligotrophic, as described above (Fierer et al., 2007, 2012), nitrogen fertilization increased the relative abundance of *Actinobacteriota* (**Figure 1A**), while decreasing that of *Acidobacteriota* (**Figure 1D**). The LEfSe results showed similar N-driven differences in relative abundances only for the prokaryotic genus *Sphingomonas* (**Figure 3B**). These nitrogen effects were reflected in prokaryotic diversity metrics: Shannon diversity increased at intermediate N rate (N45), with no further increase at N90 (**Table 3**), and β-diversity responded in a similar pattern (**Figure 3A**).

In contrast, supplemental N decreased arylsulfatase activity (**Figure 6D**), whereas the intermediate N rate (N45) resulted in the highest N-acetyl-β-glucosaminidase activity (**Figure 6B**). In addition to direct N effects on soil microorganisms, N fertilization can stimulate plant growth, thereby increasing root mass (both living and dead), altering root exudate composition, and modifying plant-microbial interactions in the soil (Zhu et al., 2016). A study using a model plant *Arabidopsis thaliana* showed that plants can modulate their root exudation chemistry and microbial relations to cater to the nutrient demand over the growth stages (Zhao et al., 2021). The reason why an enzyme that mediates sulfur cycling was negatively affected by nitrogen is unclear. By contrast, N-acetyl-β-glucosaminidase mediates nitrogen cycling, and our results suggest that the N45 rate alleviated microbial N limitation, whereas at the higher N rate (N90), soil N availability was sufficient, reducing the need for microbes to invest energy in acquiring N from organic sources via N-acetyl-β-glucosaminidase activity.

### 4.4 Relationships among soil microbial communities and soil enzymatic activities

Prokaryotes were involved in the cycling of carbon, nitrogen, phosphorus, and sulfur, but fungi were mostly involved in cycling of carbon and, to a lesser extent, sulfur. The significant role of soil carbon in enzyme activities is further illustrated by the positive correlations of the C-, N-, and P-cycling enzymes with the soil POXC (**Supplementary Table 6**). Thus, there were multiple correlations (positive or negative) between the relative abundances of the six most abundant prokaryotic genera and the activities of all four enzymes (**Table 5**). For fungal genera, comparable results were observed for the C cycling enzyme β-glucosidase, while they exhibited weaker correlations with the other enzymes. Fungi play a major role in the breakdown of recalcitrant plant residues in the soil because they have lower metabolic nutrient demand and a broader suite of extracellular enzymes than bacteria (Wardle et al., 2004).

Although the α-diversities of prokaryotes (**Table 3**) and fungi (**Table 4**) differed significantly among some treatments, the enzyme activities (**Figure 6**) did not show corresponding differences. This mismatch indicates that high structural diversity does not necessarily translate into stronger functional activity. Such functional redundancy arising from high microbial diversity and community complexity is beneficial for ecosystem functioning, because it enhances resilience when certain microbial taxa are lost under inclement environmental conditions (Wagg et al., 2019).

The inclusion of perennial forage crops into annual crop rotations likely enhanced biological soil health through microbial processes, including biological nitrogen fixation by rhizobia, nutrient cycling, mycorrhiza-facilitated nutrient uptake, biological pest suppression, and agrochemical detoxification. These improvement in soil function may have contributed to the superior agronomic and economic performance of perennial-based cropping sequences previously reported from the same experiment (Khanal et al., 2021; Bhattarai et al., 2026). Those studies consistently demonstrated better performance of creeping red fescue-based cropping sequences across one to multiple rotation cycles compared with annual crop sequences. Relative to wheat-canola rotation, intermittent inclusion of red clover and meadow bromegrass in the cropping sequences resulted in up to a 108% increase in canola equivalent yield (calculated as price ratio of non-canola to canola seed multiplied by non-canola yield) and up to a 77% increase in agronomic nitrogen used efficiency (Bhattarai et al., 2026). In the North China Plain, diversifying the traditional simple wheat-maize rotations by adding sweet potato, peanut, and soybean increased equivalent yield by up to 38% and reduced N_2_O emissions by 39% (Yang et al., 2024). Similarly, modeling of farm survey results in North Spain showed that increasing forage productivity had potential to mitigate yield-scaled greenhouse emissions, while grass-clover rotations provided additional benefits such as reduced N fertilizer costs and increased soil carbon (Doltra et al., 2018). However, in a succession of land uses from agriculture to forestry in Michigan, USA, Levine et al. (2011) found no relationship between total soil bacterial diversity and CO_2_ emissions. Although we did not measure greenhouse gas emissions to link them with the soil microbial properties in this study evidence from other studies reports mixed outcomes, highlighting the importance of including this aspect in future investigations.

## 5 Conclusion

Integrating forage seed crops in the sequential succession of annual grain crops improved biological soil health, as shown by increases in MBC (17%), β-glucosidase activity (22%), and fungal richness (e.g., Chao1 indices of 92.8 vs. 87.6). These results support our hypothesis that integrating perennial forage seed crops into annual crop sequences would enhance soil microbial communities. However, the second part of the hypothesis that legume forage crops would outperform grass crops in terms of biological soil health indicators was not supported by our results: the opposite trend was observed. Whereas fungi were associated primarily with C cycling, prokaryotes were associated with C, N, P and S cycling, highlighting the broader functional roles of prokaryotic communities. Therefore, crop sequences composed solely of annual grain crops should be diversified with perennial forage seed crops to improve soil health. An additional benefit of including perennials in the cropping systems in northwestern Canada would be revival of the dwindling forage seed industry, which has declined as many forage seed producers switched to simplified annual crop rotations, thereby compromising soil health. In this study, integrating perennial forage seed crops, particularly creeping red fescue, into the annual cropping systems improved biological soil health. Further research should investigate the microbial communities at deeper soil layers to assess the influence of deep-rooted perennials relative to annual crops and should include a common test crop across all crop sequences to link soil microbial differences with ecosystem services such as greenhouse gas mitigation, nutrient recycling, and nutrient uptake.

## Data Availability

The raw sequence reads data for Accession PRJNA1259003 is available under ID 1259003 - BioProject - NCBI (https://www.ncbi.nlm.nih.gov/bioproject/PRJNA1259003). The raw data supporting the conclusions of this article will be made available by the authors, without undue reservation.
