# Correction: Soil microbial communities shift in response to cropping sequence diversification with perennial seed crops

**DOI:** 10.3389/frmbi.2026.1934327

**Published:** 2026-07-30

**Authors:** Newton Z. Lupwayi, Nityananda Khanal, Mathew Richards, Rodrigo Ortega Polo

**Affiliations:** 1 Lethbridge Research and Development Centre, Agriculture and Agri-Food Canada, Lethbridge, AB, Canada; 2 Beaverlodge Research Farm, Agriculture and Agri-Food Canada, Beaverlodge, AB, Canada

**Keywords:** annual crops, crop rotation, nitrogen fertilizer, perennial crops, soil enzyme activities, soil health, soil microbial diversity

The copyright for this article was incorrectly written as “Copyright ^©^ 2026 Lupwayi, Khanal, Richards and Ortega Polo. This is an open-access article distributed under the terms of the Creative Commons Attribution License (CC BY). The use, distribution or reproduction in other forums is permitted, provided the original author(s) and the copyright owner(s) are credited and that the original publication in this journal is cited, in accordance with accepted academic practice. No use, distribution or reproduction is permitted which does not comply with these terms.”

The correct statement is “Copyright © 2026 His Majesty the King in Right of Canada, as represented by the Minister of Agriculture and Agri-Food for the contribution of Newton Z. Lupwayi, Nityananda Khanal, Mathew Richards, and Rodrigo Ortega Polo. This is an open-access article distributed under the terms of the Creative Commons Attribution License (CC BY). The use, distribution or reproduction in other forums is permitted, provided the original author(s) and the copyright owner(s) are credited and that the original publication in this journal is cited, in accordance with accepted academic practice. No use, distribution or reproduction is permitted which does not comply with these terms.”

The original version of this article has been updated.

