# Supplementary material for: Soil microbial communities shift in response to cropping sequence diversification with perennial seed crops
Source: Front Microbiomes. 2026 May 26;5:1808609. doi: 10.3389/frmbi.2026.1808609 (PMC13246705; doi:10.3389/frmbi.2026.1808609)
Supplement: Supplementary file 1 [file Supplementaryfile1.docx]

TABLE S1. Summary of responses^a^ of prokaryotic phyla relative abundances (RAs) to crop sequencies and nitrogen fertilizer. Only the phyla with mean relative abundances of at least 1% are included.

| Prokaryotic phylum | Mean RA (%) | Crop sequence effect (Highest; Lowest) | Nitrogen effect (Highest; Lowest) | Interaction |
| --- | --- | --- | --- | --- |
| *Actinobacteriota* | 28.35 | ** (S4; S7) | ** (90; 0) | NS |
| *Proteobacteria* | 22.45 | * (S8; S2) | NS | NS |
| *Chloroflexi* | 10.70 | * (S5; S7) | NS | NS |
| *Acidobacteriota* | 9.31 | * (S7; S6) | ** (0; 90) | NS |
| *Bacteroidota* | 8.95 | NS | NS | NS |
| *Crenarchaeota* | 7.94 | * (S2; S8) | NS | NS |
| *Planctomycetota* | 2.54 | * (S7; S6) | ** (0; 90) | NS |
| *Cyanobacteria* | 2.37 | ** (S8; S4) | * (90; 0) | NS |
| *Firmicutes* | 2.16 | ** (S4; S1) | ** (90; 0) | NS |
| *Gemmatimonadota* | 2.04 | NS | ** (0; 90) | NS |
| *Verrucomicrobiota* | 1.96 | ** (S7; S5) | ** (0; 90) | NS |

^a^NS = not significant at 5% significance level; * = significant at 5% significance level; ** = significant at 1% significance level.

TABLE S2. Summary of responses^a^ of prokaryotic genera relative abundances (RAs) to crop sequencies and nitrogen fertilizer. Only the genera with mean relative abundances (RA) of at least 1% are included.

| Prokaryotic genus (Phylum) | Mean RA (%) | Crop sequence effect (Highest; Lowest) | Nitrogen effect (Highest; Lowest) | Interaction |
| --- | --- | --- | --- | --- |
| *Sphingomonas* (*Proteobacteria*) | 5.59 | ** (S8; S7) | ** (90; 0) | NS |
| *Marmoricola* (*Actinomycetota*) | 4.42 | ** (S5; S7) | ** (90; 0) | NS |
| *Nocardioides* (*Actinomycetota*) | 4.09 | * (S4; S7) | * (90; 0) | NS |
| *C0119* (*Chloroflexi*) | 3.69 | NS | NS | NS |
| *Flavisolibacter* (*Bactereidota*) | 2.38 | NS | NS | NS |
| *Chloroplast* (*Cyanobacteria*) | 2.37 | ** (S8; S4) | * (90; 0) | NS |
| *JG30_KF_CM45* (*Chloroflexi*) | 2.17 | NS | ** (90; 0) | NS |
| *Clostridium_sensu_stricto_13* (*Firmicutes*) | 1.64 | ** (S4; S3) | * (90; 0) | NS |
| *Segetibacter* (*Bactereidota*) | 1.62 | NS | * (90; 0) | NS |
| *67_14* (Actinobacteriota) | 1.40 | * (S4; S3) | NS | NS |
| *Massilia* (*Proteobacteria*) | 1.36 | ** (S8; S1) | NS | NS |
| *WD2101_soil_group* (*Planctomycetota*) | 1.32 | * (S7; S6) | ** (0; 90) | NS |
| *Pseudolabrys* (*Proteobacteria*) | 1.27 | NS | NS | NS |
| *Blastococcus* (Actinobacteriota) | 1.11 | ** (S6; S7) | ** (90; 0) | NS |
| *Crossiella* (*Actinomycetota*) | 1.00 | NS | NS | NS |

^a^NS = not significant at 5% significance level; * = significant at 5% significance level; ** = significant at 1% significance level.

TABLE S3. Summary of responses^a^ of fungal class relative abundances (RAs) to crop sequencies and nitrogen fertilizer. Only the classes with mean relative abundances of at least 1% are included.

| Fungal class | Mean RA (%) | Crop sequence effect (Highest; Lowest) | Nitrogen effect | Interaction |
| --- | --- | --- | --- | --- |
| *Sordariomycetes* | 6.96 | ** (S8; S2) | NS | NS |
| *Dothideomycetes* | 5.73 | ** (S4; S1) | NS | * |
| *Tremellomycetes* | 2.71 | ** (S5; S7) | NS | NS |
| *Eurotiomycetes* | 2.57 | NS | NS | NS |
| *Mortierellomycetes* | 1.83 | NS | NS | NS |
| *Leotiomycetes* | 1.43 | ** (S3; S4) | NS | NS |

^a^NS = not significant at 5% significance level; * = significant at 5% significance level; ** = significant at 1% significance level.

Table S4. Summary of responses^a^ of fungal genera relative abundances (RAs) to crop sequencies and nitrogen fertilizer. Only the genera with mean relative abundances of at least 0.2% are included.

| Fungal genus (Class) | Mean RA (%) | Crop sequence effect (Highest; Lowest) | Nitrogen effect (Highest; Lowest) | Interaction |
| --- | --- | --- | --- | --- |
| *Naganishia* (*Tremellomycetes*) | 2.23 | ** (S2; S7) | NS | NS |
| *Knufia* (*Eurotiomycetes*) | 1.08 | NS | NS | NS |
| *Sclerostagonospora* (*Dothideomycetes*) | 0.87 | ** (S7; S1) | * (90; 0) | * |
| *Paraphoma* (*Dothideomycetes*) | 0.77 | ** (S8; S3) | NS | NS |
| *Penicillium* (*Eurotiomycetes*) | 0.49 | ** (S5; S3) | NS | NS |
| *Lecythophora* (*Sordariomycetes*) | 0.44 | ** (S7; S3) | NS | NS |
| *Cadophora* (*Leotiomycetes*) | 0.41 | NS | NS | NS |
| *Coniochaeta* (*Sordariomycetes*) | 0.37 | ** (S1; S8) | NS | NS |
| *Rhizophlyctis* (*Rhizophlyctidomycetes*) | 0.37 | ** (S7; S5) | NS | NS |
| *Pectenia* (*Leotiomycetes*) | 0.35 | * (S6; S2) | NS | NS |
| *Alternaria* (*Dothideomycetes*) | 0.26 | ** (S7; S6) | NS | NS |
| *Idriella* (*Leotiomycetes*) | 0.26 | ** (S8; S5) | NS | NS |
| *Niesslia* (*Sordariomycetes*) | 0.24 | ** (S2; S8) | NS | NS |
| *Clonostachys* (*Sordariomycetes*) | 0.23 | ** (S8; S1) | NS | NS |
| *Mortierella* (*Mortierellomycetes*) | 0.23 | NS | NS | NS |

^a^NS = not significant at 5% significance level; * = significant at 5% significance level; ** = significant at 1% significance level.

Table S5. Correlations (n = 95) of the relative abundances of the most abundant prokaryotic phyla and fungal classes (in descending order) with permanganate oxidizable carbon (POXC). Only those phyla and classes with significant correlations are listed.

| Prokaryotic phylum or fungal class | Prokaryotic lifestyle | Correlation (probability) with POXC |
| --- | --- | --- |
| Prokaryotic phylum |  |  |
| *Proteobacteria* | Copiotrophic | 0.345 (<0.001) |
| *Chloroflexi* | Oligotrophic | -0.282 (0.006) |
| *Acidobacteriota* | Oligotrophic | -0.482 (<0.001) |
| *Bacteroidota* | Copiotrophic | 0.301 (0.003) |
| *Planctomycetota* | Oligotrophic | -0.344 (<0.001) |
| Fungal class |  |  |
| *Sordariomycetes* | N/A^a^ | 0.387 (<0.001) |
| *Tremellomycetes* | N/A | -0.261 (0.011) |
| *Eurotiomycetes* | N/A | -0.443 (<0.001) |
| *Leotiomycetes* | N/A | -0.320 (0.002) |

^a^N/A = not applicable for fungi because they are not classified the same way.

Table S6. Correlations (n = 96) of enzyme activities with permanganate oxidizable C (POXC).

| Enzyme | Correlation (probability) with POXC |
| --- | --- |
| β-glucosidase | 0.433 (<0.001) |
| N*-*acetyl*-*β-glucosaminidase | 0.245 (0.016) |
| Acid phosphomonoesterase | 0.422 (<0.001) |
| Arylsulphatase | -0.060 (0.563) |
